# Investigation of *Copaifera* genus as a new source of antimycobaterial agents

**DOI:** 10.2144/fsoa-2020-0018

**Published:** 2020-05-29

**Authors:** Jéssica Aparecida Alves, Fariza Abrão, Thaís da Silva Moraes, Jaqueline Lopes Damasceno, Marcos Fernando dos Santos Moraes, Rodrigo Cassio Sola Veneziani, Sérgio Ricardo Ambrósio, Jairo Kenupp Bastos, Mayker Lázaro Dantas Miranda, Carlos Henrique Gomes Martins

**Affiliations:** 1 Research Laboratory of Applied Microbiology, University of Franca, Franca, SP, Brazil; 2 Nucleus of Research in Sciences & Technology, University de Franca, Franca, SP, Brazil; 3 School of Pharmaceutical Sciences of Ribeirão Preto, University of São Paulo, Ribeirão Preto, SP, Brazil; 4 Federal Institute of Education, Science & Technology of the Triângulo Mineiro, Uberlândia, MG, Brazil; 5 Laboratory of Research on Antimicrobial Trials (LaPEA), Institute of Biomedical Sciences – ICBIM, Federal University of Uberlândia, Uberlândia, MG, Brazil

**Keywords:** antimicrobial activity, *Copaifera* spp., crude extracts, *Mycobacterium tuberculosis*, oleoresins

## Abstract

**Aim:**

This paper reports on the antimycobacterial activity of the oleoresins and extracts obtained from *Copaifera* spp.

**Materials & methods:**

The minimum inhibitory concentration (MIC) and fractional inhibitory concentration index techniques helped to evaluate the effect of these oleoresins and extracts against six strains of mycobacteria that cause tuberculosis.

**Results & conclusion:**

Among the assayed oleoresins and plant extracts, the *Copaifera langsdorffii*, *Copaifera duckei*, *Copaifera reticulata* and *Copaifera trapezifolia* oleoresins provided the lowest MIC values against some of the tested strains. The combination of *Copaifera* spp. samples with isoniazid did not evidence any synergistic action. Some *Copaifera* spp. oleoresins may represent a future source for the discovery of new antimycobacterial drugs due to their low MIC values.

## Lay abstract

The purpose of this paper was to evaluate the antimycobacterial activity of the oleoresins and extracts obtained from *Copaifera* spp. against a panel of bacteria capable of causing tuberculosis. After completion of the experiments we concluded that oleoresins are better antimicrobial agents than extracts against evaluated bacteria and may represent a future source for the discovery of new antimycobacterial drugs.

## Graphical abstract

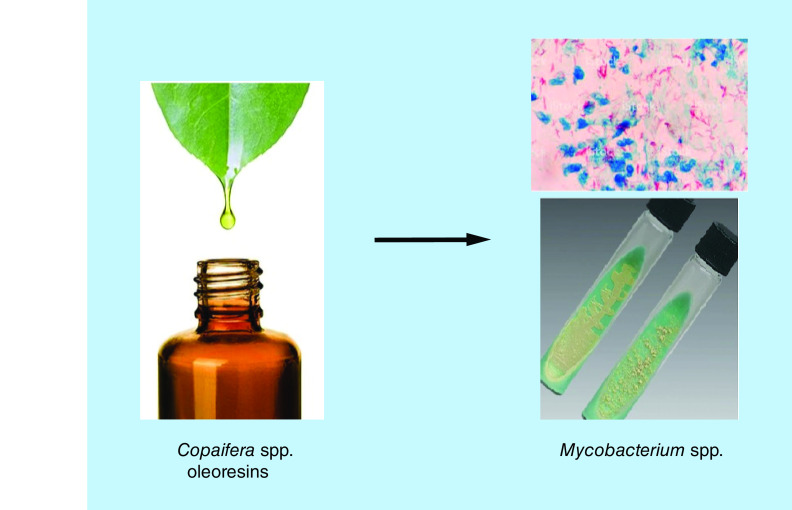

Tuberculosis (TB) is one of the oldest diseases known to humankind. It affects and kills thousands of people worldwide. The *Mycobacterium* genus causes this disease and *Mycobacterium tuberculosis* is the main species accounting for TB in humans. Pulmonary TB is the most common manifestation of the disease, but it can affect other regions of the body [1,2]. There are several obstacles to the control of TB, including difficulties and delays in diagnosis, drug resistance, lengthy treatment regimens, lack of a highly efficacious vaccine and incomplete understanding of what controls disease transmission, infectivity, reactivation and progression [3,4].

The WHO claims that TB is one of the top ten causes of death in the world. In 2018, an estimated 10 million people developed TB and 1.5 million died from the disease. Approximately 500,000 new cases of multidrug-2 and rifampicin-resistant TB are estimated to emerge annually; only one in three cases were reported to have been treated in 2018 [5]. Multidrug-2 and rifampicin-resistant TB is characterized by resistance to at least rifampicin and isoniazid, the two most powerful drugs against TB, used in standard first-line treatment [6,7]. In addition, adverse reactions, multi-drug resistance and long treatment periods make it difficult for patients to comply with TB therapy. Possible co-infection of patients with HIV/TB reinforces the need to find new therapeutic agents to treat TB [6].

Nontuberculous mycobacteria comprise more than 160 bacterial species, including the *Mycobacterium avium* complex and *Mycobacterium kansasii*, some of which may cause disease in humans. Chronic pulmonary infection is the most common clinical manifestation. Although patients suffering from chronic lung diseases are particularly susceptible to nontuberculous mycobacteria pulmonary disease, many affected patients have no apparent risk factors [2].

Indiscriminate use of antimicrobials and potential bacterial recombination have given rise to resistant pathogenic microorganisms, which has made various commercially available drugs ineffective. This scenario has stimulated the search for new therapeutic agents derived from plant species [7–11]. Medicinal plants have long been used to treat several infectious diseases. These plants produce numerous compounds with biological activity, so that they can represent an important source of new antimicrobial agents for drug discovery and development [12–18]. Moreover, higher plant extracts have been considered as promising sources of novel anti-TB leads [19].

The *Copaifera* genus stands out for its huge variety of secondary metabolites. Copaiba oleoresin is a transparent liquid with variable color and viscosity. Studies on Copaibeiras have revealed that sesquiterpenes and diterpenes are the main compounds in the oleoresin and extracts obtained from these plants [20–22]. The use of *Copaifera* spp. in folk medicine is diverse, which indicates that these plants display countless pharmacological properties, including significant potential anti-inflammatory, analgesic and antimicrobial action [23]. The antimicrobial activity of the oils obtained from different *Copaifera* species in Brazil have made them a promising source for the discovery of new and selective agents for the treatment of major infectious diseases [24–33].

Specifically, *Copaifera langsdorffii* Desf. (Fabacecae-Ceasalpinoidae), popularly known as ‘copaiba’, is a native species of tropical regions of Latin America and is widely distributed in the Brazilian Cerrado and other forests. The oleoresin has a strong antioxidant and anti-inflammatory effects and is composed of several sesquiterpenes and unsaturated diterpene acids [34]. The species *Copaifera duckei* Dwyer belongs to the family Fabaceae, which produces an oleoresin that comes from the exudation of the trunk of the tree. In folk medicine, traditional communities use this oleoresin through oral or topical administration for various medicinal purposes. Several indications have been described, including wound healing, anti-inflammatory action and antigastric and antiulcer effect, among other digestive diseases [35]. *Copaifera reticulata* is a medium tree known as ‘copaibeira’ and ‘pau d’óleo’. It is native to tropical regions of south America and grows abundantly in several states of Brazil, like Pará, Amazonas and Ceará. The oleoresin of this plant has been used mainly as healing, anti-inflammatory and antiseptic agent. The main chemical constituents of this oleoresin are mono-, sesqui- and diterpenes; many of its biological activities can be attributed to these compounds [36]. In turn, *Copaifera trapezifolia* Hayne, a species that is easily found in the Brazilian south-eastern region, is noted for its economic and medicinal importance [37,38]. Leandro *et al.* [20] investigated the antibacterial activity of the hydroalcoholic extract obtained from *C. trapezifolia* leaves (CTE) against microorganisms that cause dental caries and apical periodontitis, to find that *C. trapezifolia* leaves exhibits antibacterial activity against some of the tested microorganisms under the employed experimental conditions. The authors emphasized that this plant species can be an important source of biologically active compounds and aid in the search for new effective and safe agents that can act against oral pathogens.

Considering the pharmacological potentials of Brazilian *Copaifera* species [39], this study aimed to evaluate the antimycobacterial activity of the oleoresins and hydroalcoholic crude extracts obtained from *Copaifera* spp. leaves against some mycobacterial species and the interaction between compounds from this plant species and the antibiotic isoniazid.

## Materials & methods

### Plant material & chemical characterization

*Copaifera* leaves and oleoresins (when available) were collected in different states, between August 2012 and May 2014, which was authorized by the Brazilian government through SISBIO (35143-1) and CGEN (010225/2014-5). Table 1 contains information about voucher specimens. To extract the oleoresins, a hole of approximately 1 inch wide and 3 feet above the ground was made into the center of the trunk of the tree with an auger. The oleoresin was drained into an amber bottle by using a pipe connected to a filter and the hole was then properly sealed [40]. The leaf extracts were prepared as described in Furtado *et al.* [41].

**Table 1.** Collection information concerning the *Copaifera* species.

*Copaifera* species	Location (city/state)	Herbarium	Identification number
*Copaifera langsdorffii*	Cajuru/SP	SPFR^†^	14438
*Copaifera duckei*	Belém/PA	EMBRAPA^‡^	175206
*Copaifera reticulata*	Brasil Novo/PA	EMBRAPA	175266
*Copaifera multijuga*	Manacapuru/AM	EMBRAPA	180069
*Copaifera lucens*	Rio de Janeiro/RJ	JBRJ^§^	474303
*Copaifera oblongifolia*	Pirajuba/MG	SPFR	14437
*Copaifera trapezifolia*	Rio de Janeiro/RJ	EMBRAPA	178494

† SPFR: Herbarium of the Department of Biology, Faculty of Philosophy, Sciences and Letters of Ribeirão Preto, University of São Paulo (SPFR), Brazil.

‡ EMBRAPA: Brazilian Agricultural Research Corporation (Embrapa Eastern Amazon), Brazil.

§ JBRJ: Rio de Janeiro Botanical Garden, Brazil.

### Microorganisms

To evaluate the antimycobacterial activity of the oleoresins and the hydroalcoholic crude extracts from *Copaifera* spp., the following American Type Culture Collection (ATCC) mycobacteria and clinical isolates (were kindly supplied by Adolfo Lutz Institute) were used: *M. tuberculosis* H37Rv (ATCC 27294), *M. tuberculosis* (clinical isolate CF 16), *M. kansasii* (ATCC 12478), *M. kansasii* (clinical isolate 2776), *M. avium* (ATCC 25291) and *M. avium* complex (clinical isolate 2781).

### Determination of MIC

To evaluate the antimycobacterial activity of the samples, the MIC was determined by microdilution in a microplate. Resazurin was used to reveal bacterial growth by the resazurin microtiter assay (REMA) method procedure adapted from Palomino *et al.* [42]. The experiment was conducted in triplicate.

The oleoresins and the hydroalcoholic crude extracts were dissolved in DMSO and serially diluted in Middlebrook 7H9 broth before inoculation. The concentrations of the tested compounds ranged from 31.25 to 2000 μg ml^-1^. Isoniazid, dissolved in DMSO, was used as standard drug at concentrations ranging from 0.015 to 1.0 μg ml^-1^. The inoculum was prepared by introducing a range of colonies grown in Ogawa–Kudoh (LaborClin) in a tube containing glass beads with 500 μl of sterile water. A 200-μl aliquot was transferred to a tube containing 2 ml of 7H9 broth, incubated at 37°C for 7 days and compared with McFarland scale 1. After the inoculum was standardized, it was diluted with 7H9 broth at a 1:25 ratio. Growth controls containing no antibiotic and sterility controls without inoculation were also included. The plates were incubated at 37°C for 7 days. Subsequently, 30 μl of 0.02% resazurin aqueous solution was added to each well. After standing for 18 h, a blue color (coloring resazurin solution) was interpreted as lack of bacterial growth, whereas a pink color indicated the presence of viable micro-organisms. MIC was defined as the lowest drug concentration that was able to inhibit 90% growth of the mycobacterium strain. The assay was conducted in triplicate.

### Determination of the fractional inhibitory concentration index

The activities of combinations of the oleoresins and hydroalcoholic crude extracts with isoniazid were evaluated by fractional inhibitory concentration index (FICI) for each of the tested mycobacteria. Checkerboard assays were performed according to the protocol previously described by Bhusal *et al.* [43] with modifications to investigate the *in vitro* antimicrobial efficacy of the combination of the compounds with the positive control (isoniazid). The whole experiment was repeated three-times. To determine FICI, the concentrations of oleoresins, hydroalcoholic crude extracts and isoniazid were the same as the concentrations used in the MIC assay. The bacterial inoculum was also employed at the same concentration and the inoculum was prepared in the same way described in the section about the MIC assay.

Serial twofold dilutions of compounds and isoniazid were mixed in each well of a 96-well microtiter plate. Fifty-microliter aliquots of the antimicrobial agent (oleoresins or extracts) at an initial concentration of 8000 μg ml^-1^ were added in vertical and horizontal orientation, respectively. Next, 100 ml of 1 × 10^8^ CFU/ml fresh bacterial suspension was added to each well and incubated as mentioned previously. After 7 days of incubation at 37°C, resazurin was added to each well to reveal bacterial growth.

FICIs were calculated according to the following formula: FICI = (MIC of antimicrobial agent in combination with isoniazid/MIC of antimicrobial agent alone) + (MIC of isoniazid in combination with antimicrobial agent/MIC of isoniazid alone). Synergism was defined as FICI ≤0.5; additive effect was defined as 0.5 < FICI <1; indifference was defined as 1 ≤ FICI <4 and antagonism was defined as FICI ≥ 4 [44].

## Results & discussion

Silva *et al.* [40] and in Furtado *et al.* [41] described the chemical characterization of all the oleoresins and leaf extracts. Here, we have investigated the antimycobacterial activity of the oleoresins and hydroalcoholic crude extracts obtained from the leaves of *Copaifera* spp. by determining the MIC and the FICI. Table 2 summarizes the MIC values determined for the oleoresins and the hydroalcoholic extracts obtained from the leaves of *Copaifera* spp. against the tested mycobacteria.

**Table 2.** MIC - μg ml^-1^ results obtained for the oleoresins and hydroalcoholic crude extracts obtained from *Copaifera* spp. against mycobacteria.

Extract/oleoresin/PC	*Mycobacterium avium* - I.C 2781	*Mycobacterium avium* - ATCC 25291	*Mycobacterium kansasii - I.C* 2776	*Mycobacterium kansasii* - ATCC 12478	*Mycobacterium tuberculosis* – H37Rv ATCC 27294	*Mycobacterium tuberculosis I.C CF 16*
*Copaifera trapezifolia* – extract	1000	1000	>2000	1000	1000	500
*Copaifera oblongifolia* – extract	1000	500	>2000	500	250	500
*Copaiferaduckei* – extract	1000	500	>2000	1000	125	500
*Copaifera lucens* – extract	1000	500	1000	250	125	500
*Copaifera langsdorffii* – oleoresin	500	125	125	62.5	250	125
*Copaifera duckei* – oleoresin	500	62.5	125	62.5	62.5	125
*Copaifera reticulata* – oleoresin	125	125	125	250	500	62.5
*Copaifera oblongifolia* – oleoresin	125	125	500	500	250	125
*Copaifera multijuga* – oleoresin	>2000	2000	1000	1000	250	125
*C. trapezifolia* – oleoresin	1000	500	125	500	125	62.5
Isoniazid (PC)	1.0	>1.0	1.0	1.0	0.6	1.0

ATCC: American Type Culture Collection; PC: Positive control.

MIC ranged from 62.5 μg ml^-1^ to values above 2000 μg ml^-1^. According to Holetz *et al.* [45], crude extracts with MIC values lower than 100 μg ml^-1^, between 100 and 500 μg ml^-1^, from above 500 to 1000 μg ml^-1^ and greater than 1000 μg ml^-1^ display good antimicrobial activity, moderate antibacterial activity, weak antibacterial activity and absence of antibacterial activity, respectively. Other authors have considered that MICs ≤200 μg ml^-1^ for extracts indicate good activity against *M. tuberculosis* [46].

The *C. duckei* oleoresin displayed MIC of 62.5 μg ml^-1^ against *M. avium* (ATCC 25291), *M. kansasii* (ATCC 12478) and *M. tuberculosis* (ATCC 27294). The same results were found for the *C. langsdorffii* oleoresin against *M. kansasii* (ATCC 12478) and the *C. reticulata* oleoresin against *M. tuberculosis* (clinical isolate CF16). These results demonstrated the great potential of these oleoresins against some mycobacterial species.

Based on the criteria of Holetz *et al.* [45], these results corresponded to good antimycobacterial activity and demonstrated that compounds from natural products are an important source of lead drugs for the research and development of new antimycobacterial agents.

The hydroalcoholic extracts obtained from *C. duckei*, *Chionanthus lucens*, *C. langsdorffii*, *C. duckei*, *C. reticulata*, *Copaifera oblongifolia*, *C. trapezifolia* and *Copaifera multijuga* leaves displayed MIC of 125 μg ml^-1^ against the mycobacteria *M. kansasii* (clinical isolate), *M. tuberculosis* (ATCC and clinical isolate) and *M. avium* (ATCC and clinical isolate). The hydroalcoholic extracts obtained from *C. lucens* and *C. oblongifolia* leaves and the oleoresins obtained from *C. langsdorffii*, *C. reticulata*, *C. oblongifolia* and *C. multijuga* leaves presented MIC of 250 μg ml^-1^ against *M. tuberculosis* (ATCC) and *M. kansasii* (ATCC). The hydroalcoholic extracts obtained from *C. trapezifolia*, *C. oblongifolia*, *C. duckei* and *C. lucens* leaves and the oleoresins obtained from *C. langsdorffii*, *C. duckei*, *C. reticulata*, *C. oblongifolia* and *C. trapezifolia* leaves exhibited MIC of 500 μg ml^-1^ against *M. kansasii* (ATCC and clinical isolate), *M. tuberculosis* (ATCC and clinical isolate) and *M. avium* (ATCC and clinical isolate). These results were considered moderate for antimycobacterial activity.

Natural antimicrobial products may lead to different results. The differences observed between the oleoresins and the hydroalcoholic extracts obtained from leaves herein might stem from the distinct composition of the oleoresins and the extracts. From a chemical viewpoint, *Copaifera* oleoresins comprise a diterpene acid solution in an essential oil consisting mainly of sesquiterpenes [40,41,47]. Currently, over 70 sequiterpenes have been identified in different *Copaifera* species. From a biological standpoint, the oleoresin consists of a waste product or detoxification plant organism and acts to protect the plant against animals, fungi and bacteria [48]. Additionally, phytochemical studies on these plants have concluded that the hydroalcoholic crude extracts obtained from ‘copaiba’ leaves are rich in polar compounds, which can be extracted by aqueous alcohol extraction. Thus, Furtado *et al.* [41] have recently described the presence of phenolic compounds like flavonoid heterosides and galloylquinic acid derivatives in such extracts. The quercetin flavonoids and kaempferol-3-*O*-α-L-rhamnopyranoside substances were first described in the hydroalcoholic extract of the aerial parts of *C. landsgorffii*. This class of substances is well known for their different biological activities, especially their anticancer and antioxidant actions [49]. According to Sikkema *et al.* [50], an important feature accounting for the antimicrobial activity of oils is the presence of hydrophobic lipid components that can partition the bacterial cell membrane, thereby disintegrating structures and making them more permeable.

In most cases herein, the crude hydroalcoholic extracts obtained from the leaves proved to be less effective in fighting bacteria as seen in the MIC results. This is because the oleoresins contain higher concentration of active molecules than the crude hydroalcoholic extracts obtained from the leaves [23].

In this study, the MIC results that were chosen to determine the FICI values were less than or equal to 125 μg ml^-1^, these MIC values gave the best results and inhibited mycobacterial growth the most during the tests. The oleoresins and the hydroalcoholic crude extracts that provided MIC values in this range were the *C. trapezifolia*, *C. duckei*, *C. langsdorffii*, *C. reticulata*, *C. oblongifolia* and *C. multijuga* oleoresins and the extracts obtained from *C. duckei* and *C. lucens*.

The FICI results obtained after evaluation of the synergistic action between the oleoresins or the hydroalcoholic crude extracts and isoniazid against each of the tested mycobacteria did not evidence any synergism. Indeed, the results were antagonistic or indifferent (data not shown). Even though our data did not support any synergy between the oleoresins or extracts and isoniazid, we investigated the combination of these compounds in an attempt to improve the antimycobacterial activity. Hochfellner *et al.* [51] evaluated the interaction between quinolone and indoloquinazoline alkaloids with rifampicin or isoniazid against *Mycobacterium smegmatis* (ATCC 14468). The authors did not find satisfactory interaction between the evaluated molecules and rifampicin or isoniazid.

According to Van *et al.* [52], antagonistic interactions should not be ignored when investigating interactions in plant-based studies. These authors reported that the increasing number of scientific works exploring interactions among herbal substances is encouraging and relevant in our ongoing quest to understand the mechanisms of action of herbal medicine.

It is worth mentioning that the oils and extracts used in the MIC tests and in combination with isoniazid herein were weighed against a group of bacteria that are difficult to treat because they have cell wall and different proteins. Bearing this in mind, we can consider that the MIC values obtained herein are promising. Not to overlook possible associations of the substances tested herein with different drugs, we shall conduct future studies using a greater variety of drug combinations, which will hopefully result in new treatment alternatives.

Moreover, the literature contains few studies on the use of *Copaifera* against mycobacteria, another reason this study is important to guide future research in this field.

## Conclusion

The oleoresins obtained from *C. langsdorffii*, *C. duckei*, *C. reticulata* and *C. trapezifolia* inhibited the growth of the mycobacteria *M. kansasii* (ATCC), *M. tuberculosis* (ATCC and clinical isolate) and *M. avium* (ATCC) satisfactorily. The FICI showed that the combination of one of the extracts or oleoresins tested herein with isoniazid exerted antagonistic or indifferent effects on the tested mycobacteria.

In conclusion, the results of this study, especially the MIC values lower than 100 μg ml^-1^, suggested that investigation into the properties of the oleoresins and extracts obtained from some *Copaifera* species could contribute to expanding scientific knowledge about the antimycobacterial activity of this plant genus and aid the discovery of new antimycobacterial drugs.

## Future perspective

Although TB has become a treatable disease thanks to the emergence of antibiotics, it is still a difficult disease to treat – treatment lasts long and a large amount of antibiotics is used by the patient. Increased bacterial resistance is a reality in which scientists seek to discover new drugs that can fight infection with minimal damage to the patient. Existing drugs have a large number of side effects and should be replaced with safe molecules. In this context, medicinal plants have been explored as sources of molecules with promising antibacterial activity. Few studies involving antimycobacterial activity and Brazilian medicinal plants have resulted in satisfactory effects. The oleoresins used in this study proved to be safe in the toxicity tests conducted by our research group. The oleoresins evaluated here are promising in the search for new antimycobacterial compounds. It is necessary to understand their mechanism of action and to undertake further studies on molecules obtained from *Copaifera* spp. oleoresins.

The results suggest that the oleoresins obtained from some *Copaifera* species can contribute to the discovery of new antimycobacterial drugs, but studies involving its mechanism of action should be undertaken.

**Executive summary**

- The *Copaifera langsdorffii*, *Copaifera duckei*, *Citrus reticulata*, *Copaifera multijuga* and *Copaifera trapezifolia* oleoresins were effective against the *Mycobacterium* spp evaluated in this study.
- Among the investigated *Copaifera* species, *C. duckei* displayed the best antimycobacterial results.
- Oleoresins in the absence of isoniazid were more effective.
